# Intrauterine Transmission of Hepatitis C Virus Concomitant with Isolated Severe Fetal Ascites

**DOI:** 10.3390/pathogens11111335

**Published:** 2022-11-12

**Authors:** Cristiana Luiza Rădoi, Elena-Iuliana-Anamaria Berbecaru, Anca-Maria Istrate-Ofițeru, Rodica Daniela Nagy, Roxana Cristina Drăgușin, Razvan Grigoraș Căpitănescu, Marian Valentin Zorilă, Lucian George Zorilă, Dominic Gabriel Iliescu

**Affiliations:** 1 Doctoral School, University of Medicine and Pharmacy of Craiova, 200349 Craiova, Romania; 2 Department of Obstetrics and Gynecology, Emergency Clinical County Hospital, 200642 Craiova, Romania; 3 Department of Histology, University of Medicine and Pharmacy of Craiova, 200349 Craiova, Romania; 4 Research Centre for Microscopic Morphology and Immunology, University of Medicine and Pharmacy of Craiova, 200349 Craiova, Romania; 5 Department of Obstetrics and Gynecology, University of Medicine and Pharmacy of Craiova, 200349 Craiova, Romania; 6 Department of Forensic Medicine, University of Medicine and Pharmacy of Craiova, 200349 Craiova, Romania

**Keywords:** fetal infections, fetal ascites, congenital hepatitis, prenatal diagnosis, ultrasound, amniocentesis, fetal paracentesis, cordocentesis

## Abstract

Background: Perinatal Hepatitis C Virus (HCV) transmission occurs in 4–7% of the cases with detectable viremia at delivery. HCV testing in pregnancy is recommended. The fetal infection was previously described as asymptomatic although there are two cases, including this one, to report the presence of isolated fetal ascites in HCV infected fetuses. Case report: A 42-year-old patient, 3G, 3P, presented in the Emergency Room for painful uterine contraction. The third-trimester ultrasound examination noted severe fetal ascites, accompanied by hyperechoic bowels and polyhydramnios. The diagnosis required a detailed ultrasound exam, invasive testing (amniocentesis, cordocentesis, and fetal paracentesis), and a complete workup. The mother tested positive for HCV antibodies, and the fetal cord blood tested positive for HCV RNA. The ascites resolved after paracentesis, and the gastrointestinal and respiratory functions markedly improved. The fetus was delivered at term in good condition. Conclusions: The etiology of isolated fetal ascites is broad. This case may indicate that intrauterine HCV transmission is a potential cause of isolated fetal ascites in the absence of other explanation, and isolated fetal ascites can be the only sign revealed on a routine examination. We suspected, having no other detected cause for ascites, the intrauterine transmission of HCV. Invasive procedures, such as paracentesis, are required for abdominal decompression to manage isolated fetal ascites, as it may be a saving procedure. A genetic investigation is needed, and a good neonatal outcome is expected in the absence of fetal structural or genetic abnormalities, as in our case.

## 1. Introduction and Literature Review

### 1.1. Congenital Hepatitis C Virus

Hepatitis C virus (HCV) infection is a global health issue, affecting 2–3% of the worldwide population. The declared highest infection rate is in Northern Africa, almost 3%, and the lowest is in Northern Europe, under 1%. The CDC (Centers for Disease Control and Prevention) recommends HCV testing for all pregnant women. Unfortunately, there is no antiviral treatment approved during pregnancy yet. However, HCV screening allows for appropriate assessment of liver disease status and improves the follow-up for infants at risk of vertical transmission [1,2]. 

Acute HCV infection develops in the first six months after the virus exposure and is usually asymptomatic. When symptoms occur, they are not specific and involve jaundice, anorexia, nausea, and abdominal pain [3,4]. Although 15–45% of the patients usually clear the virus in the first six months, those who do not will develop chronic infection. After 20 years of infection, the patients with chronic disease have a high risk for cirrhosis and hepatocellular carcinoma [5,6].

HCV is a bloodborne infection. The main risk factors for HCV transmission are blood products transfusions, transplantation of solid organs, injecting drug use, unsafe therapeutic injections, occupational exposure to blood, and unprotected sexual intercourse with an infected partner [1,7,8,9,10]. Data on the effect of virus concentration are inconsistent, but the transmission mostly appears to consistently increase in levels of HCV-RNA above 106 IU per mL [11,12,13,14]. Vertical transmission or mother-to-child transmission of HCV may occur intrauterine, at delivery, or in the first 28 days after birth. The mechanisms of mother-to-child transmission (MTCT) are not well understood, but it is stated that HCV monoinfected pregnant women have a 2–8% risk of viral transmission to their infant [15]. 

As expected, mothers’ intravenous drug use has an increased vertical transmission rate compared to women who have not been involved in such practices [16,17]. In addition, associated infections play an important role in HCV transmission. HIV co-infection increases the transmission rate two-fold [11,18,19]. Other authors declare even higher rates [15,20,21]. However, recent studies are showing an additional risk of HCV transmission in the setting of maternal HIV co-infection – this risk is reduced if maternal HIV is well controlled with antiretroviral therapy (ART) [22,23,24]. Peripheral blood mononuclear cell infection with HCV is associated with a higher risk of vertical transmission, possibly as a vector for HCV [25,26]. 

The relation between the mode of delivery and HCV perinatal transmission is controversial. Some authors have suggested that with vaginal delivery, vertical HCV transmission increases due to an increased risk of exposure to virus-contaminated maternal blood. Therefore, cesarean section was proposed as a safer option [27]. Four large studies were carried out to evaluate the HCV transmission risk with elective cesarean versus vaginal or emergency cesarean section [13,28,29,30]. Two studies reported a higher transmission risk with vaginal delivery or emergency caesarian section than in the cases with planned caesarian delivery, which was statistically significant in only one of them [29,30]. Other studies, including the European Pediatric Hepatitis C Virus Network study, reported that delivery mode does not appear to influence transmission rate [15,28,31,32,33].

As a general concept, maternal age, mode of delivery, parity, HCV genotype, and breastfeeding (if nipples are not cracked or bleeding) do not appear to be risk factors for HCV vertical transmission [7,20,21,34].

Clinics and perinatal treatment. HCV infection transmitted vertically is usually asymptomatic in infancy and is associated with elevated ALT (alanine aminotransferase) levels in the first years of life. At the moment, there are few long-term follow-up studies on the natural history of HCV infection in children and adolescents and no long-term studies to evaluate if the vertically transmitted HCV progresses into hepatic failure or hepatocellular carcinoma [13,35,36,37,38,39]. Elevated ALT levels (in 1/3 of the children) and hepatomegaly (in 10%) were the most common changes reported [13]. Most of the studied children did not obtain a spontaneous clearance in those cases (92%), and some of them (1.8%) even progressed to decompensated cirrhosis [35]. 

The HCV screening strategy aims to identify and treat all persons HCV-positive before conception [10].

There are no treatment options recommended during pregnancy. IFN (interferon) alpha is considered category C class risk during pregnancy [15,40]. Ribavirin is considered category X, and treatment is forbidden during pregnancy or in couples (also for male partners) who intend to obtain a pregnancy for at least 6 months before conception [18]. 

A newly developed treatment, direct-acting antivirals (DAAs), has replaced IFN regimens. The advances in HCV treatment do not apply during pregnancy [15]. A small phase 1 study evaluating the pharmacokinetics of ledipasvir-sofosbuvir in pregnancy reported 100% SVR12 (sustained virological response 12 weeks after completion of treatment) and no safety concerns [41]. Recently, the FDA approved DAA regimens for children after 3 years old, for any HCV genotype [38]. 

Children born from HCV-positive mothers are at risk of contracting HCV intrauterine, intrapartum, or after delivery. Therefore, they should be tested for HCV infection. In addition, ¼ to ½ of infected infants obtain a spontaneous clearance of the infection in the first 4 years [36,38,42,43,44,45,46].

The diagnosis of HCV infection in children born from HCV mothers has become important due to new studies and guidelines that allow HCV treatment for children as young as three years old [38]. Anti-HCV antibodies pass the placental barrier from the mother’s blood to the fetal bloodstream and may persist in the infant’s blood until 18 months. HCV-RNA can be determined by PCR (polymerase chain reaction). This test is more expensive but allows an earlier diagnosis of HCV infection in infants. Still, this type of very early diagnosis cannot be proved useful because treatment options are available only after the age of 3 [38,47]. 

### 1.2. Fetal Ascites

Fetal ascites is usually diagnosed by ultrasound examination. It is always considered an abnormal finding, and the etiology must be investigated. Fetal ascites may appear isolated or associated with organic malformations, hydrops, infections, genetic syndromes, and other conditions [48,49]. 

Fetal hydrops is usually associated with pericardial effusion, pleural effusion, and skin edema. When these signs are missing, an isolated fetal ascites is assumed. It is essential to differentiate the two because the prognosis and management are significantly different [50]. When associated with hydrops and respiratory tract malformation, the fetal prognosis is mainly unfavorable, with a high risk of fetal or neonatal death [48]. 

Perinatal prognosis of fetal ascites is better when isolated. A study of 79 cases of nonimmune fetal ascites showed a 57% mortality. This study involved 25 (31.6%) isolated fetal ascites. The survival rate was reported to be higher for the fetuses with isolated fetal ascites (49.2%), compared with a 33% survival rate for the cases with hydrops fetalis [51]. Secondary to stretching, the newborn may present diastasis of rectus abdominalis muscles and redundant abdominal skin [52]. 

When investigating the etiology of fetal ascites, a systematic protocol must be followed [49,53]. The workup should include the mother Rhesus, infection screening, fetal karyotyping, and detailed ultrasound examination to establish if other congenital abnormalities are associated [53]. When paracentesis is performed, the evaluation of serum-ascites albumin gradient (SAAG) may be used to discriminate between transudate and exudate and differentiate the possible causes of fetal ascites. If SAAG is greater than 1.1 g/L, the ascites may be caused by portal hypertension; otherwise, it may have a non-portal hypertension etiology [54]. 

The assessment of fetal ascites may Ide various biological screening such as maternal isoimmune antibodies, feto-maternal hemorrhage, fetal anemia, fetal hemoglobinemia, glucose-6-phosphate deficiency, thalassemia, and infections (syphilis, toxoplasmosis, cytomegalovirus, parvovirus, and coxsackievirus). In addition, ultrasound evaluation for fetal malformations and genetic tests (chromosomal analysis and fetal karyotype) are also necessary. Other causes of fetal ascites may be chylous ascites, meconium peritonitis, and urinary peritonitis. According to a recent metanalysis involving 315 cases of isolated fetal ascites, the etiology was genitourinary (24%), gastrointestinal (20%), viral or bacterial infections (9%), cardiac (9%), genetic disorders (8%), chylous ascites (6%), metabolic storage disorders (3%), other structural disorders (4%), other causes (4%), and idiopathic (13%) [55]. 

Chromosomal abnormalities can be associated with fetal ascites; therefore, prenatal genetic testing is essential. Chorionic villus sampling and amniocentesis are used for the genetic assessment, but they can also help diagnose fetal infections and inherited metabolic disease [50,52]. 

Few cases are reporting the association between isolated fetal ascites and viral infections. Hepatitis A is not usually involved in congenital infections. We found 2 cases reporting hepatitis A virus associated with fetal ascites and meconium peritonitis. Both were surgically treated after delivery [56,57,58]. One case of HCV infection associated with isolated fetal ascites in the second trimester of pregnancy was reported. A paracentesis was performed, and a favorable outcome was reported [59]. In addition, one case of Hepatitis E virus infection associated with fetal ascites was reported. In this case, the ascites resolved spontaneously during pregnancy, and the fetus had a good outcome [58].

Hyperechoic fetal bowel is a soft ultrasound genetic marker that may be associated with fetal aneuploidies, especially Trisomy 21 [60,61], but also with small bowel obstruction, oligohydramnios [62], Hirschsprung disease, bowel atresia, intrauterine growth restriction [63], intraamniotic hemorrhage [64], cystic fibrosis [65,66], maternal infection with CMV, Toxoplasmosis, Parvovirus B19 [64], or HCV [59]. In many cases, it may resolve itself, with normal bowel function, in most newborns [60,67,68,69]. The pathogenic mechanism of hyperechogenic bowel may be explained by hypoperistalsis, decreased fluid content of the meconium [60,70], or chronic intrauterine bowel ischemia [60,71,72]. Another cause of the hyperechoic aspect of the bowel is meconium peritonitis, which is a sterile inflammatory reaction of the peritoneum caused by in utero bowel perforation with intraperitoneal extravasation of the meconium [50,73,74,75]. It is a rare cause of peritonitis, often fatal [76], that may be caused by ischemia in the mesentery, volvulus, intestinal atresia, meconium plugs, internal hernia, Hirschsprung’s disease, colon atresia, torsion of a fallopian tube, and cystic fibrosis. In addition to the echogenic aspect of the bowel, meconium peritonitis may be suspected during the ultrasound examination in the presence of polyhydramnios (100%), bowel dilatation (53%), ascites (33%), and pseudocyst (13%) [77]. 

## 2. Case Report

A 42-year-old patient, gravida 3, para 3, with an obstetrical history of two vaginal births and a personal history of congenital deaf-mute, with no surgical history or blood transfusions, was admitted to our clinic for painful uterine contractions with an onset of approximately 24 h. The patient had no previous antenatal obstetrical care.

**Figure 1:**
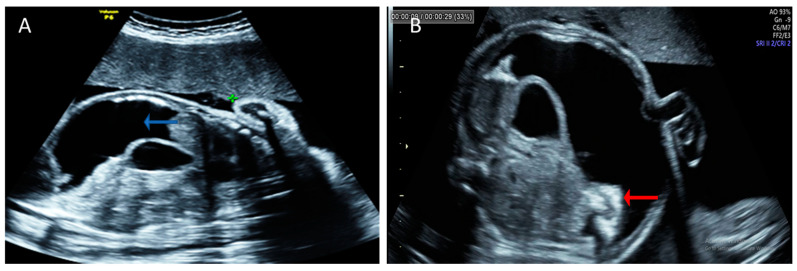
Initial fetal evaluation with severe ascites. (**A**) Longitudinal view of the fetal abdomen showing severe ascites (blue arrow). (**B**) Hyperechoic aspect of the bowel, with caudal and posterior displacement (red arrow).

**Figure 2:**
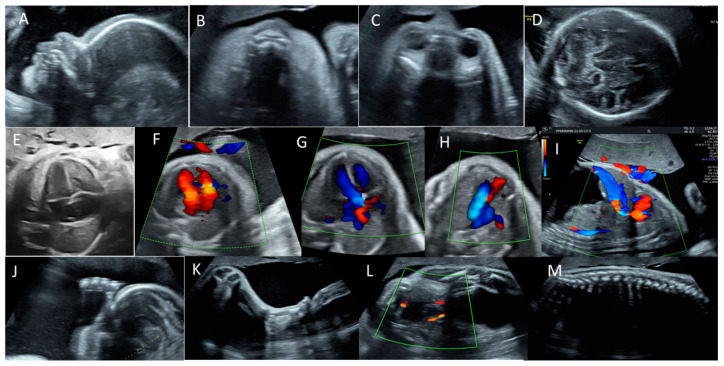
Features of the anomaly scan, where no other fetal anomalies were detected. (**A**) Facial profile. (**B**) Anterior palate. (**C**) Axial orbital plane. (**D**) Cerebellum and thalami. (**E**–**H**) Normal features of the heart: four-chamber Iw (**E**), atrio-ventricular flows (**F**), left ventricular outflow tract (**G**), and three-vessel and trachea view (**H**). (**I**) Presence of ductus venosus. (**J**,**K**) Normal aspects of the limbs. (**L**) Bladder, with two umbilical arteries aside. (**M**) Normal aspect of spine.

The ultrasound examination showed 31 weeks and 4 days of fetal biometry with a marked discordance between the abdominal circumference measurements and the rest of the biometrics due to massive fetal ascites. Therefore, we only used head circumference, biparietal diameter, and long bones measurements for gestational age evaluation. Hyperechoic fetal bowels (Figure 1) and polyhydramnios were noted, with an AFI (amniotic fluid index) of 28 cm. The fetal breathing was present but very limited due to the severe ascites. The Middle Cerebral Artery (MCA) and Umbilical Artery (UA) Doppler assessments were normal. There were no signs of other fetal structural abnormalities (Figure 2).

Further tests were planned to detect the cause of fetal ascites. The blood count, and liver and kidney function were found normal. The patient’s fasting blood sugar level was monitored daily. The values varied in a normal range (78–106 mg/dL) with 5.89% HbA1c.

The genetic counseling recommended invasive testing.

The extended TORCH complex revealed high titers of IgG antibodies for Cytomegalovirus (CMV), Herpes Simplex type 1 and 2, Toxoplasmosis, Epstein Barr, Parvovirus B19, and Rubella, with negative IgM antibodies for all the above.

The patient was also tested for HBV, H½ HIV 1/2 infections, and Syphilis. The anti-HCV antibodies were positive in the mother’s blood (44.55 cut-off-index (COI)). 

The HCV infection of the mother associated with fetal ascites, hyperechoic bowels, and polyhydramnios guided the investigations for potential transplacental transmission of HCV, but we could not dismiss other causes.

A therapeutic paracentesis was planned to improve the fetal prognosis by abdominal decompression and to help investigate the etiology of fetal ascites.

At the beginning of the procedure, cordocentesis was performed under ultrasound guidance for fetal anesthesia and to retain fetal blood samples for further assay. The umbilical cord was punctured at the placental insertion site, and 8 mL of fetal blood was collected from the umbilical vein. Then, to reduce fetal movements during paracentesis, anesthetics were injected according to recent guidelines [59]. The fetal blood was tested for blood count, albumin, bilirubin levels, blood sugar level, the direct Coombs test, HCV RNA, CMV IgM, Toxoplasmosis IgM, Rubella IgM, and Herpes Simplex type 1 and 2 IgM.

**Figure 3:**
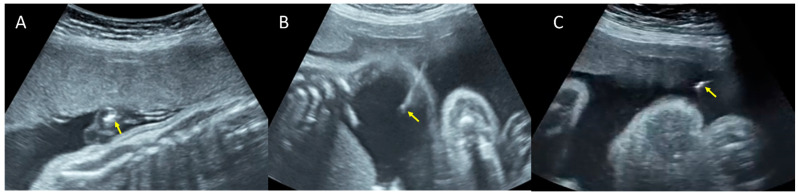
Ultrasound-guided multiple invasive procedures. Arrows are pointing to the tip of the needle: (**A**) Cordocentesis. (**B**) Paracentesis. (**C**) Amniocentesis.

Under ultrasound guidance, an 18 G needle was inserted in the fetal peritoneal cavity for paracentesis, and 160 mL of clear fluid was extracted until ascites became minimal (Figure 3). Cytological and biochemical analyses were performed (leukocytes, red blood cells, glucose, albumin, creatinine, urea, proteins, Rivalta reaction, cytological examination, and blood smear).

Following paracentesis, an amniocentesis was also performed, and 40 mL of clear amniotic fluid was collected for further tests (viral DNA for herpes simplex, Epstein Barr, and parvovirus).

After the maneuvers, both the mother and the fetus were stable, with an optimal fetal biophysical profile score (BPS). In addition, fetal breathing movements were visibly improved. 

Fetal blood investigation revealed normal blood count and a negative Direct Coombs test. The fetus tested negative for CMV, Toxoplasmosis, Rubeola, Herpes simpl½type 1/2, Epstein Barr, and Parvovirus B19. The anti-HCV antibodies were absent, but the HCV RNA was detectable (251 UI/mL). The mother HCV RNA was determined on the same day by PCR (polymerase chain reaction) with a 2,365,000 UI/mL result.

Fetal ascites assay: The cytological exam had no clinical significance. The Serum Ascites Albumin Gradient (SAAG) was >1.1 g/dL, so the ascites was considered transudate. The rest of the biological tests from ascites fluid were within normal ranges.

**Figure 4:**
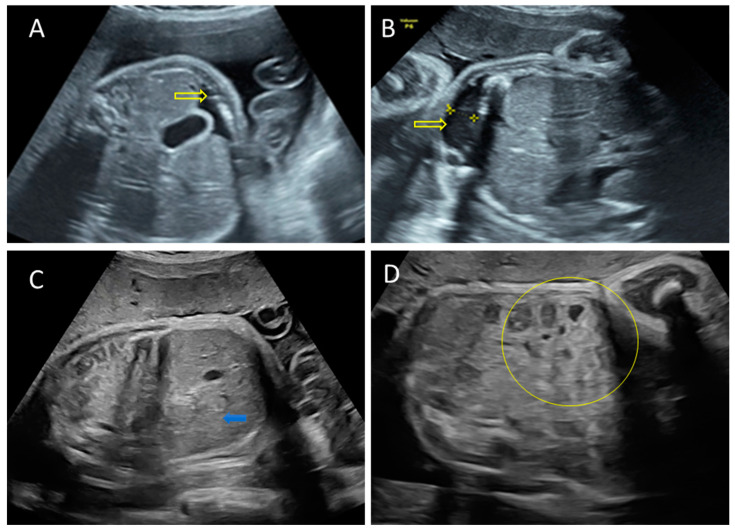
Ascites remission following paracentesis. (**A**,**B**) Minimal fetal ascites immediately after paracentesis (arrows). (**C**) Mild hepatomegaly is noted (blue arrow). (**D**) Absent fetal ascites in the first week after procedure. We highlight the normal intestinal echogenicity (circle).

Two days after paracentesis, the ultrasound exam showed normal amniotic fluid levels, fetal ascites remission, and mild hepato-splenomegaly (Figure 4). 

During the hospitalization, the pregnancy was carefully monitored. The fetus maintained normal AFI and BPS.

No signs of fetal structural abnormalities were detected. Thus, we considered that intrauterine transmission of HCV was the cause of fetal ascites, hyperechogenic bowels, and polyhydramnios.

We could not establish the mother infection moment or transmission pattern. In the literature, the primary risk factor for HCV transmission was considered to be transfusion of blood and blood products, but for our patient, we can only consider a blood product transfusion before HCV testing was introduced as a screening method in general transfusion practice. We could not gather this kind of information from her or from family. Injection, drug use, or sexual contact may be more probable, as a lack of prenatal care reflects an underlying high-risk social situation. None of these conditions can be highlighted as infection factors for this case. We do not know the infection status of the sexual partner, and from the declared history or clinical examination, there were no signs of drug use.

The positive fetal blood HCV-RNA and the ultrasonographic aspect suggested a decompensated hepatitis C of the fetus.

Patient parenteral/oral hepatoprotective treatment was administered, as there was no possibility of antiviral treatment at that moment. The patient was discharged from the hospital and examined weekly. The ascites remained absent, AFI was noted within normal ranges until birth, and the bowels echogenicity appeared normal.

**Figure 5:**
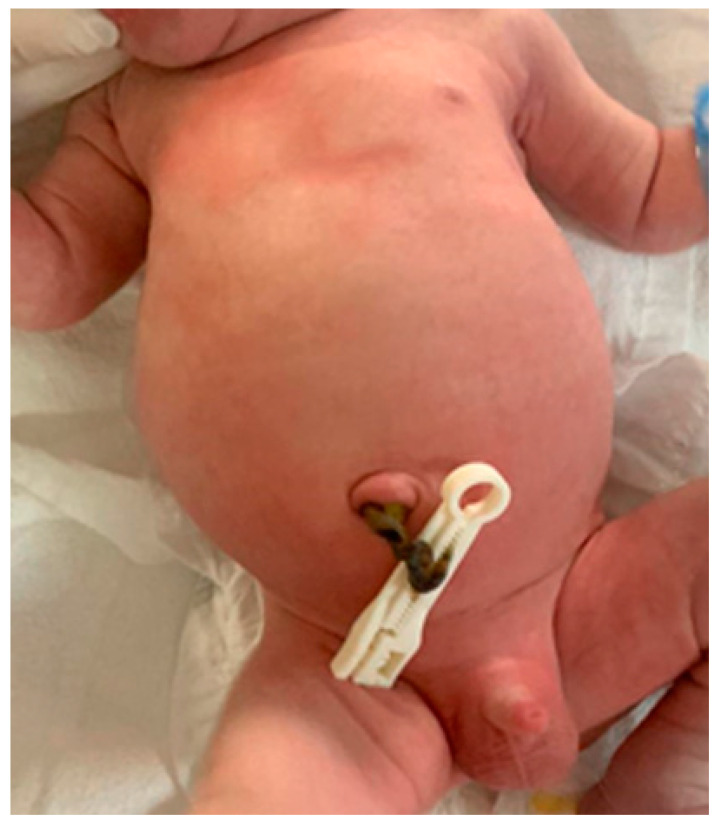
Newborn normal aspect with a slight abdominal skin excess.

Five weeks later, the patient was admitted in advanced labor and delivered vaginally a male fetus weighing 2880 g, with an Apgar Score of 7 and no signs of respiratory distress. Fetal ascites was absent, and a slight abdominal skin excess was noted (Figure 5). After the delivery, the mother and the newborn had a good evolution and were discharged after three days. A pediatric exam was recommended to establish a proper postnatal monitoring and therapeutic plan.

The newborn liver function and blood cell count were normal, with present anti-HCV antibodies. The infant continued the follow-up exams. At the age of 3 months, the anti-HCV antibodies were present, and HCV RNA was detected. At 9 months, the anti-HCV antibodies persisted, and the HCV RNA was still measurable

## 3. Discussion

Fetal ascites can easily be diagnosed by ultrasound examination and should be weekly monitored to determine a potential deterioration of the fetal condition, with progression to hydrops [50]. Following repeated ultrasound examinations, in our case, the fetus did not show other signs of hydrops, so we assumed isolated fetal ascites. Isolated fetal ascites can be caused by urinary tract obstruction, meconium peritonitis, primary lymphangiectasia, laryngeal atresia or stenosis, chromosomal abnormalities, viral or infections, hepatic insufficiency in storage diseases, or it can be idiopathic [51]. Even though the prognosis of isolated fetal ascites is better than in hydrops cases, the etiological diagnosis is challenging, usually requiring invasive testing [50]. Genetic disorders can cause fetal ascites [53,55]; therefore, the fetal karyotype was determined and chromosomal anomalies were dismissed. Most of the cases are associated with other anomalies, so an exhaustive work-up must be followed [49,53]. The first ultrasound assessment showed fetal ascites associated with hyperechoic bowels and polyhydramnios and no other structural anomaly. Moreover, the hyperechogenic bowel aspect subsided, and the echogenicity appeared normal after abdominal decompression.

The diIgnosis of intrauterine transmission of HCV was established following the combined fetal and maternal biological assay. We ruled out a potential isoimmune fetal hydrops because the mother’s Rh was positive. On fetal blood samples, bilirubin levels were in the normal range, the Coombs test was negative, and fetal anemia was not suspected at the ultrasound scan (peak systolic velocity of the middle cerebral artery).

After performing a detailed maternal screening for infections, we found anti-HCV antibodies present and over 2 mil UI/mL copies of HCV-RNA in the mother’s blood, which reportedly increases the risk of intrauterine transmission of HCV [12,13,14]. Therefore, our investigations focused on determining if the fetal ascites may be explained by a MTCT (Mother-to-Child Transmission) of HCV in utero. We found detectable HCV-RNA in the fetal cord blood sample (>250 UI/mL) without anti-HCV antibodies, which may suggest a recent HCV infection of the fetus. Obtaining a fetal blood sample did not require additional maneuvers, because cordocentesis is necessary to perform fetal anesthesia for paracentesis. Otherwise, the diagnosis of intrauterine MTCT is not mandatory, as no treatment is available until the age of 3 years; thus, postpartum assessment can be postponed. As a major discussion topic, there is an undetermined risk of vertical transmission during intrauterine invasive procedures, especially cordocentesis (the risk analysis was not well studied).

Severe fetal ascites compresses the diaphragm, mediastinum, and abdominal viscera, thus restricting deglutition and intestinal transit, and leading to polyhydramnios. Moreover, bowel compression determines hypoxia and the secondary hyperechoic aspect. When possible, a paracentesis should be performed for decompression of the abdominal viscera and diagnostic tests, allowing the intestines and the diaphragm to resume a normal position and development [78]. To improve the fetal outcome, we performed a fetal paracentesis with complete resolution of the ascites in 4 weeks. El Bishry et al. monitored 12 cases of isolated fetal ascites and reported complete resolution of fetal ascites after paracentesis in 50% of the cases (30% antepartum and 20% after birth). Reducing the diaphragm compression on the lungs by paracentesis allows for proper pulmonary development and function after birth [78]. A paracentesis may be repeated in cases when the asIites level increases again. An abdominal-amniotic shunt may be placed to avoid repeated paracentesis [79,80]. In our case, the ascites subsided after paracentesis and did not require further paracentesis or abdominal-amniotic shunt for decompression.

Polyhydramnios is known to be a high-risk factor for perinatal and maternal morbidity and mortality by intrauterine fetal demise, preterm labor, premature rupture of membranes, umbilical cord prolapse, transverse or breech presentation, cesarean delivery, and postpartum hemorrhage [81]. With complete resolutions of the ascites, the fetal intraabdominal pressure was reduced, and the gastrointestinal function improved.

Therefore, the amniotic fluid quantity subsided, reducing the risk of preterm birth. The patient delivered vaginally at term, a fetus with normal respiratory and digestive function. The mother and child were discharged two days after birth.

The maternal HCV-IgG antibodies may persist in the infant’s blood until 18 months of life. AASLD/IDSA (American Association for the Study of Liver Diseases/Infectious Diseases Society of America) recommends antibody tests at or after 18 months of age or an HCV RNA test after 2 months of age for diagnosis [38,47]. Furthermore, if the child is anti-HCV-antibodies-positive after 18 months, he should be tested for the presence of HCV-RNA after 3 years to confirm a chronic hepatitis C infection [38]. We confirmed the presence of HCV RNA in fetal blood and the HCV antibodies seropositivity after delivery and at 3 months of age. HCV RNA was also detected at 9 months of age. The patient had no prolonged rupture of membrane or cracked nipples that may have exposed the fetus to further HCV viral load. Therefore, the only explanation for the persistent infection remains in utero transmission of HCV.

The patient did not attend obstetrical visits during pregnancy or periconceptional counseling. The diagnosis of HCV infection before pregnancy allows the treatment of both partners before conception, reducing the risk of MTCT. Antiviral therapies available are safe and well-tolerated and have the potential to cure this chronic infection before pregnancy in almost 95% of those treated, reducing the risk of cirrhosis, liver cancer, and further liver failure [2,82]. Currently, there is no antiviral treatment approved for HCV during pregnancy or for newborns [2,38]. To our knowledge, there is only one case reporting intrauterine or peripartum transmission of HCV associated with isolated fetal ascites and polyhydramnios.

## 4. Conclusions

Maternal chronic hepatitis C infection associated with isolated fetal ascites is rare. However, this may be the primary and only sign of HCV intrauterine transmission.

Isolated fetal ascites is easy to diagnose, but the etiologic analysis algorithm may be challenging, as many causes are involved. Therefore, a systematic ultrasound exam, invasive fetal testing, infections assay, and highly trained physicians are required. The intrauterine transmission of HCV can be confirmed by determining the HCV-RNA in fetal blood and PCR HCV-RNA testing after 2–6 months with reliable results.

Fetal paracentesis is mandatory in managing massive isolated fetal ascites. Frequently, it is the only treatment available before birth as a rescue maneuver that facilitates fetal abdominal viscera development and polyhydramnios remission.

When available, a specific postpartum follow-up is necessary to differentiate the clearance or progression of the disease.

This is the first reported case of documented HCV RNA detection by cordocentesis in the setting of isolated fetal ascites, possibly indicating that intrauterine HCV infection was the cause of the ascites. Additional studies are necessary to investigate whether HCV vertical transmission is truly associated with a higher risk of fetal ascites.

## Data Availability

Not applicable.
